# C16-Functionalized Diatomaceous Earth: A Sustainable Approach for the Selective Encapsulation and Remediation of Hydrocarbons from Water

**DOI:** 10.3390/ma19081529

**Published:** 2026-04-10

**Authors:** Rosalia Maria Cigala, Mario Samperi, Paola Cardiano, Alessandro Tripodo, Giuseppe Sabatino, Catia Cannilla, Giuseppina La Ganga, Ileana Ielo

**Affiliations:** 1 Department of Chemical, Biological, Pharmaceutical and Environmental Science, University of Messina, Via F. Stagno d’Alcontres 31, 98166 Messina, Italy; rosaliamaria.cigala@unime.it (R.M.C.); paola.cardiano@unime.it (P.C.); giuseppina.laganga@unime.it (G.L.G.); 2 National Research Council-Institute of Advanced for Energy Technology (CNR-ITAE), Via Comunale S. Lucia 5, 98126 Messina, Italy; mario.samperi@cnr.it (M.S.); catia.cannilla@cnr.it (C.C.); 3 Department of Mathematical and Computer Sciences, Physical Sciences and Earth Sciences, University of Messina, Via F. Stagno d’Alcontres 31, 98166 Messina, Italy; alessandro.tripodo@unime.it (A.T.); giuseppe.sabatino@unime.it (G.S.); 4 National Institute of Oceanography and Experimental Geophysics, Via dei Mille 28, 98057 Milazzo, Italy; 5 Interuniversitary Research Center for Artificial Photosynthesis (SOLARCHEM), University of Messina, Via F. Stagno d’Alcontres 31, 98166 Messina, Italy

**Keywords:** diatomaceous earth, surface functionalization, oil–water separation, hydrophobicity, diesel encapsulation, sustainable remediation

## Abstract

The primary objective of this research is to engineer a high-performance, sustainable material for aquatic remediation by repurposing low-cost biogenic silica into a selective hydrophobic adsorbent. By integrating the natural hierarchical porosity of Diatomaceous Earth (DE) with a tailored silanization strategy, this work aims to provide a scalable and eco-friendly solution for the efficient encapsulation and mechanical recovery of hydrocarbons from contaminated water. To overcome the inherent hydrophilicity of DE, a two-step functionalization process was developed, involving alkaline activation followed by the covalent grafting of hexadecyltrimethoxysilane (C16) in different concentrations. The resulting C16@DE hybrid materials underwent a dramatic surface energy transformation, shifting from hydrophilic behavior to robust hydrophobicity, with static contact angles reaching up to 134.8°. Optical analysis revealed a unique remediation mechanism: while pristine DE disperses homogeneously in the aqueous phase, functionalized C16@DE spontaneously organizes into discrete pellets upon contact with diesel, effectively encapsulating the fuel. Quantitative UV/vis spectrophotometry confirmed that these composites sequester approximately 55–56% of the diesel phase. Together, these results demonstrate that C16@DE materials couple intrinsic biosilica porosity with tailored hydrophobicity to achieve efficient hydrocarbon capture. By combining the natural hierarchical porosity of diatoms with engineered surface selectivity, this research positions functionalized DE as a scalable, low-cost, and eco-friendly promising solution for marine oil spill recovery and industrial wastewater treatment.

## 1. Introduction

The development of advanced materials for environmental remediation has become a critical area of research due to the increasing frequency of industrial accidents and oil spills [1]. Specifically, addressing the global challenge of hydrocarbon contamination in aquatic environments is paramount to preserving marine biodiversity and ensuring the safety of water resources [2]. Among various naturally occurring minerals, Diatomaceous earth (DE), has emerged as a highly promising candidate for these applications because of its abundance, low cost, and unique biological origin [3,4]. DE consists of the fossilized skeletons, or frustules, of unicellular algae called diatoms, which offer structural advantages that are difficult to replicate synthetically [5]. Primarily consisting of hydrated amorphous silica, DE is characterized by a lightweight and highly porous structure [6]. It features a unique hierarchical micro-nano architecture that includes intricate pore networks and well-preserved centric frustules [7,8]. While these properties grant DE a high specific surface area and significant thermal resistance, the material is naturally hydrophilic [9,10]. This behavior is due to a surface densely populated with reactive silanol groups (-Si-OH) that interact strongly with water molecules [11,12,13]. Specifically, the literature data for pristine DE report a BET specific surface area of 3.81 ± 0.01 m^2^ g^−1^ and a total pore volume of 0.0155 ± 0.0009 cm^3^ g^−1^. These textural characteristics, along with a particle porosity of 0.043 ± 0.004 cm^3^ g^−1^, confirm its nature as a macroporous material suitable for the physical entrapment of organic molecules [14]. Consequently, to utilize DE effectively for the selective adsorption of oil and hydrocarbons from water, its surface must be chemically modified to transition from a hydrophilic state to a hydrophobic or even superhydrophobic regime [15,16,17]. Surface functionalization with low-energy chemical groups is essential for achieving durable water-repellent properties [18,19]. Among various modification strategies, the use of alkoxysilane coupling agents offers a facile and reliable pathway [20]. This process involves the formation of stable, covalent Si-O-Si bonds between the hydrolyzed silanes and the hydroxyl groups on the DE surface, ensuring long-term structural integrity [21]. Research has demonstrated that the degree of hydrophobicity is strongly dependent on the alkyl chain length of the silane precursor [22]. Specifically, it has been established that alkyl chains with 12 or more carbons (≥C12) are required to facilitate the structural ordering and molecular packing necessary for high water contact angles [23]. Hexadecyltrimethoxysilane (C16), a long-chain silane, is one of the most effective reagents for this purpose [24]. Recent studies have highlighted its effectiveness in providing a dense, ordered layer of hydrocarbon chains that effectively shields the hydrophilic silica core [25], minimizes interfacial energy and enhances selective interaction with non-polar contaminants such as diesel fuel [26,27]. By combining the intrinsic porosity of the diatom frustules with the low surface energy provided by the C16 functionalization, the resulting material can selectively capture oil molecules while repelling the aqueous phase. While various studies in the literature have explored the functionalization of DE for oil–water separation [1], primarily focusing on traditional adsorption mechanisms or the development of superhydrophobic coatings for filtration membranes [28], the novelty of this research lies in the discovery of a spontaneous “pelletization” and encapsulation mechanism. Unlike conventional DE-based sorbents that often remain dispersed in the medium or require complex secondary filtration for recovery, our C16@DE hybrid material undergoes a unique self-organization into discrete, stable granules upon contact with hydrocarbons. This behavior represents a significant departure from classical remediation paradigms, allowing for the direct mechanical recovery of encapsulated pollutants from aquatic environments. This study investigates the surface modification of DE to engineer a high-performance material for oil remediation. A systematic functionalization process was developed to graft hexadecyl (C16) chains at varying concentrations onto the DE surface. Characterization of the surface wettability revealed a significant phase transition, where functionalized samples shifted from inherent hydrophilicity to robust hydrophobicity. Additionally, the oil-adsorption capacity was evaluated using diesel-contaminated water; the C16@DE composites demonstrated an efficient encapsulation mechanism, successfully sequestering up to 56% of the fuel phase. Unlike traditional porous adsorbents, the developed C16@DE composites offer a unique dual-action remediation pathway, combining rapid surface-energy-driven oil capture with a spontaneous encapsulation into stable granules. This pelletization-driven capture process represents a notable departure from classical adsorption mechanisms. This allows for the efficient removal of hydrocarbons without the need for complex filtration, positioning functionalized DE as a sustainable and scalable solution for marine oil spill recovery.

## 2. Materials and Methods

Diatomaceous earth (DE) and hexadecyltrimethoxysilane (C16) were purchased from Sigma-Aldrich (Merck, Rahway, NJ, USA). Hydrogen peroxide (H_2_O_2_, 30%), ammonium hydroxide solution (NH_4_OH, 30%), absolute ethanol (EtOH), benzene, and distilled water (H_2_O_dist_) were purchased from VWR (Milan, Italy). The diesel fuel was obtained from a local gasoline retailer. All reagents, except for the DE, were used as received without further purification; the DE, however, underwent preliminary washing procedures as described below.

**Figure 1:**
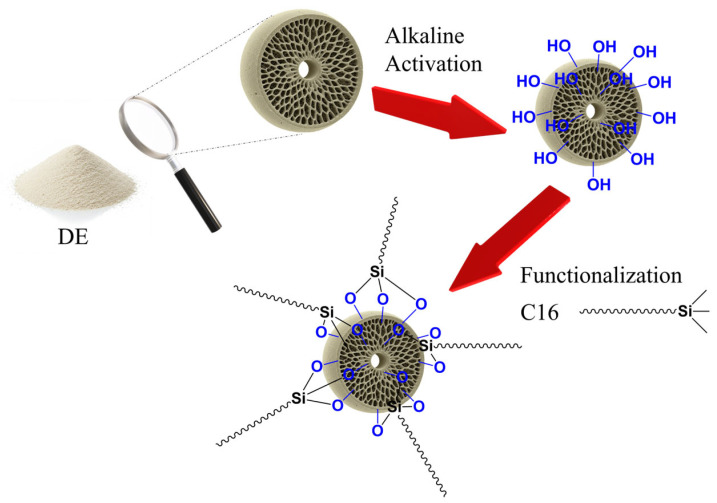
Schematic workflow of the activation and functionalization process of DE using C16 silane.

During the preparation of this manuscript, the authors used Gemini 3 Flash for the purposes of revising the English language and improving the clarity of the manuscript. Additionally, the authors used Nano Banana for the creation of part of Figure 1 and the Graphical Abstract. The authors have reviewed and edited the output and take full responsibility for the content of this publication.

### 2.1. Alkaline Activation of DE

Five 1.5 g aliquots of DE were weighed and placed in glass bottles containing 50 mL of EtOH, then treated in an ultrasonic bath for 30 min. The samples were subsequently filtered using paper filters with 0.22 μm pores, placed in beakers with 20 mL of H_2_O_dist_ and heated to 80 °C under magnetic stirring. Once the target temperature was reached, 10 mL of H_2_O_2_ and 10 mL of NH_4_OH were added to the solution. The mixture (Alkaline Piranha solution) was kept under magnetic stirring for 20 min. After this period, the DE was washed three times with abundant H_2_O_dist_, followed by a final wash with EtOH. The samples were then left to air-dry overnight. The term ‘Alkaline Piranha’ is used here to denote an NH_4_OH/H_2_O_2_ mixture analogous to RCA SC-1 cleaning solution [29].

### 2.2. Functionalization of DE with C16

Assuming the DE is composed primarily of silica (SiO_2_), the 1.5 g aliquots utilized correspond to 25 mmol of SiO_2_. Surface functionalization was performed using C16 at varying SiO_2_:C16 molar ratios, specifically 10:1, 20:1, 40:1, and 80:1. For each preparation, the appropriate stoichiometric amount of C16 was dissolved in 20 mL of EtOH. The resulting solution was combined with the activated DE powder and processed using a rotary evaporator under reduced pressure. The solvent was removed by heating the mixture to 45 °C until complete evaporation was achieved, ensuring a uniform distribution of the precursor over the DE surface. The resulting dry powder was subjected to three purification cycles consisting of washing with EtOH to remove unreacted silane species, followed by air-drying overnight. The rotary evaporation step ensured uniform precursor distribution, facilitating reproducible silanization across samples. A schematic workflow of the activation and functionalization procedures is shown in Figure 1.

### 2.3. Surface Wettability and Contact Angle Analysis

To evaluate the surface wettability and hydrophobicity of the functionalized composites, each sample was compacted into discrete disks using a Retsch PP25 manual pellet press (Retsch GmbH, Haan, Germany) under an applied pressure of 19.5 bar. Static contact angle measurements were performed via the sessile drop method; specifically, a 5 μL droplet of water was deposited onto the surface of the pellets. The interaction between the liquid and the substrate was captured using a lateral perspective (side-view) imaging setup [30]. The resulting contact angles were subsequently quantified through digital image analysis using ImageJ software (version 1.54r) and the specific contact angle plugin. Each measurement was averaged over at least three droplets placed at different positions on the pellet surface to ensure statistical reliability. Environmental conditions (temperature/humidity) were monitored to minimize variability.

### 2.4. Oil Remediation

The efficiency of the functionalized DE samples in purifying oil-contaminated water was evaluated through a diesel remediation assay. In this study, diesel fuel was selected as a representative model for hydrocarbon contamination due to its complex mixture of aliphatic and aromatic compounds, allowing for a realistic evaluation of the material’s performance in broader oil–water separation and marine remediation scenarios. For each test, 400 μL of diesel fuel was added to 100 mL of H_2_O_dist_ to simulate a contamination event. Subsequently, 200 mg of the respective functionalized or non-functionalized (control) DE powder was introduced into the mixture. The remediation process involved several separation steps: First, the DE–diesel complex was separated from the aqueous phase via filtration. To extract the residual, unadsorbed diesel from the water, 5 mL of benzene was added as an organic solvent. Benzene was specifically selected for the extraction process due to its superior solvating power for both the aliphatic and aromatic fractions of diesel fuel, combined with its high optical transparency at the analytical wavelength of 425 nm. While safer alternatives like n-hexane were considered, initial benchmarking indicated that benzene provided more reproducible extraction kinetics and a baseline-neutral environment for UV/vis quantification, which was essential for the precise determination of the sequestration capacity. To mitigate the associated safety and environmental concerns, the solvent volume was strictly minimized (5 mL per test), and all procedures were conducted under high-performance fume hoods following rigorous hazardous material handling protocols.

The mixture was then transferred to a separatory funnel, allowing for the isolation of the organic fraction containing the remaining diesel. The concentration of non-adsorbed diesel was then quantified using UV/vis spectroscopy by measuring the absorbance of the organic phase. This experimental procedure was performed systematically for all functionalized DE variants (DE_C16 10:1 to 80:1) and the pristine, non-functionalized DE to determine the relative improvement in oil uptake capacity.

### 2.5. UV/Vis Spectroscopy

UV/vis absorption spectra of all the suspensions were measured in 1 cm quartz cuvettes with a Stellarnet BLACK-Comet-SR diode array spectrophotometer (StellarNet Inc., Tampa, FL, USA) equipped with a combined deuterium/tungsten halogen lamp (Stellarnet mod. SL5) and a multimodal fiber optic cable (length: 1 m; diameter: 1 mm; optical range: 190–1100 nm).

### 2.6. ATR-FTIR

Fourier Transform Infra-red (FTIR) spectra were recorded in Attenuated Total Reflection (ATR) using a Thermo Scientific spectrophotometer model iS50ATR (Parma, Italy). The scans were performed at a resolution of 4 cm^−1^, and the spectra were reported as percentage transmittance vs. wavenumbers (cm^−1^).

### 2.7. SEM

The morphology of the samples was investigated using an Ultra-High-Resolution Scanning Electron Microscope (UHR-SEM-FEG, Helios 5 UC Dual Beam, Thermo Scientific, Parma, Italy) equipped with a Field Emission Gun (FEG), which provides high spatial resolution and improved image quality. SEM imaging was carried out on gold-coated samples using the in-lens SE/BSE detector, operating at an accelerating voltage of 10 kV and a beam current of 0.1 nA.

### 2.8. Apparent Density Determination

The density of each DE sample, including both pristine and functionalized variants, was determined following a simple and direct method based on the ratio between mass and volume, using a precision glass capillary method [31]. All measurements were performed at a controlled temperature of 25 °C. For each measurement, a glass capillary with a calibrated volume range of 1–5 μL was utilized. To ensure accuracy, the mass of the empty and filled capillary was determined via statistical weighing calculating the average over 10 independent measurements. The DE powder was introduced into the capillary and densely packed to eliminate voids and ensure a consistent volume. The volume of the sample was then quantified by measuring the length of the packed column relative to the pre-calibrated graduation marks, which indicated 1 μL increments. The bulk density (ρ) was subsequently calculated as the ratio of the sample mass to the measured volume. This procedure was systematically repeated for all functionalized and non-functionalized samples.

## 3. Results and Discussion

The chemical modification of DE was conducted via a two-step process: alkaline activation followed by silane grafting. The initial alkaline activation with Alkaline Piranha solution served to increase the density of reactive hydroxyl (–OH) groups on the silica surface. This specific activation step, analogous to the RCA SC-1 cleaning solution [29,32], was employed to remove organic impurities and hydrate the silica surface, thereby increasing the density of reactive hydroxyl (–OH) groups necessary for subsequent silanization. While silica is known to be soluble in alkaline environments, the short duration of the treatment (20 min) and the weak base used were optimized to achieve surface activation without compromising the structural integrity of the material. Indeed, SEM analysis confirmed that the hierarchical framework and the intricate pore networks of the centric frustules remained well-preserved after the treatment. The second step entails the functionalization with C16 carried out by condensation reaction between the hydrolyzed silane alkoxy groups and the surface silanols of the DE.

As the C16 concentration increased (from 80:1 to 10:1 DE:C16 molar ratios), a progressively denser organic monolayer was formed. This is expected to result in a transition from a naturally hydrophilic state to a strongly hydrophobic character, as the long alkyl chains of the silane shield the polar silica surface. The use of rotary evaporation facilitated a uniform coating, as the slow removal of ethanol under reduced pressure promotes even distribution of the precursor before covalent bonding is finalized during the drying stage.

**Figure 2:**
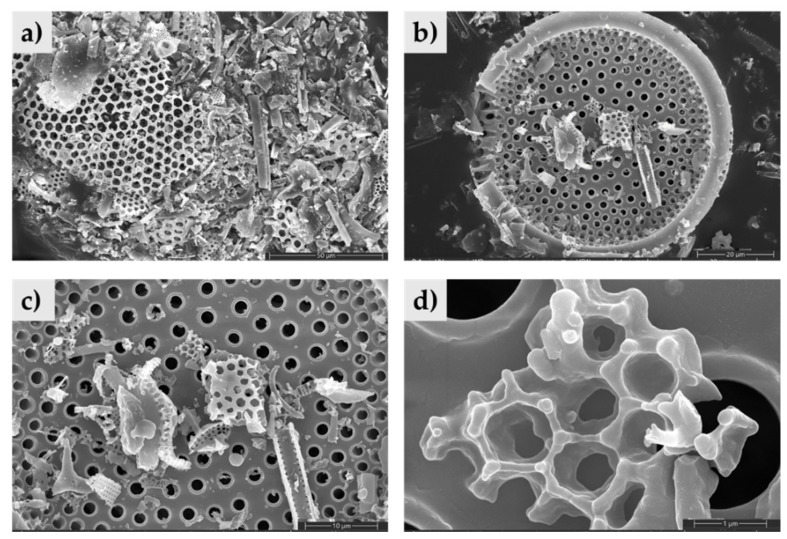
SEM micrographs of the raw DE structure at (**a**) magnification of 2.5k× and scale of 50 μm showing fossilized frustule morphology; (**b**) magnification of 4.2k× and scale of 20 μm; (**c**) magnification of 8k× and scale of 10 μm; and (**d**) magnification of 80k× and scale of 1 μm detailing the hierarchical porosity and nanometric features.

The SEM micrographs reveal a highly porous, hierarchical framework that is ideal for environmental remediation. The wide-field view in Figure 2a shows a distribution of fragmented and intact frustules. The well-preserved centric frustules (Figure 2b) and the intricate pore networks (Figure 2c) act as a robust inorganic scaffold, providing a high specific surface area available for the silanization process with C16 chains. This multiscale porosity, extending from the micro-scale areolae to the nano-scale voids seen in Figure 2d, is critical because the internal voids provide a massive geometric volume for the physical entrapment of fuel molecules. The abundance of surface hydroxyl groups on these silica structures allows for a dense grafting of C16 chains, transforming the naturally hydrophilic silica into a hydrophobic and organophilic interface.

The observed hierarchical micro-nano architecture and the well-preserved centric frustules are consistent with the structural characteristics typically reported for biogenic silica [11,25], confirming that alkaline activation and silanization processes do not compromise the robust inorganic scaffold required for effective pollutant entrapment.

**Figure 3:**
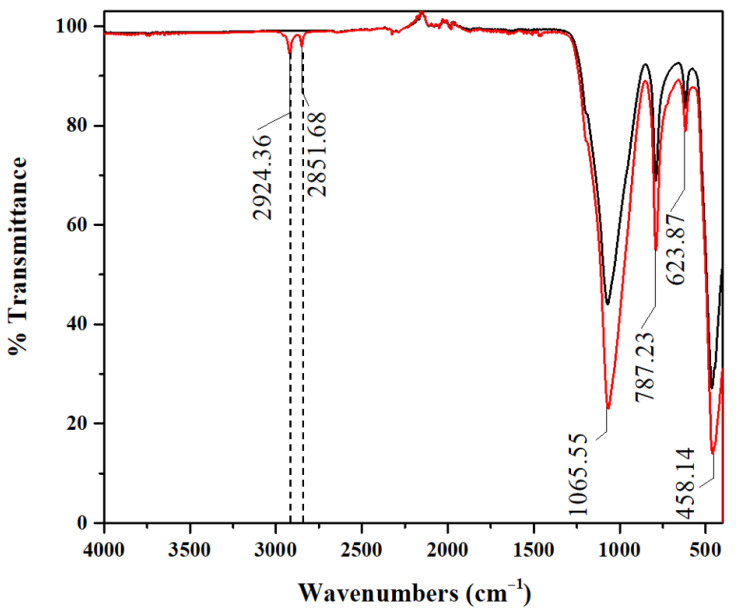
ATR_FTIR spectra of DE in black line and DE_C16 (40:1) in red line.

In Figure 3, the ATR-FTIR spectra of DE and C16@DE (40:1) are reported. The spectra overlay shows a complete reproducibility of the DE signals, asymmetric stretching of Si–O–Si at 1066 cm^−1^, symmetric stretching at 787 cm^−1^ and Si–O lattice vibrations at 623 and 458 cm^−1^. The only difference in the signals between the spectra is due to the signals at 2924 and 2851 cm^−1^ in C16@DE (40:1), which highlight asymmetric and symmetric stretching of -CH_2_-, respectively, typical of long aliphatic chains [33,34]. These last two signals, although low in intensity, show the functionalization of DE.

**Figure 4:**
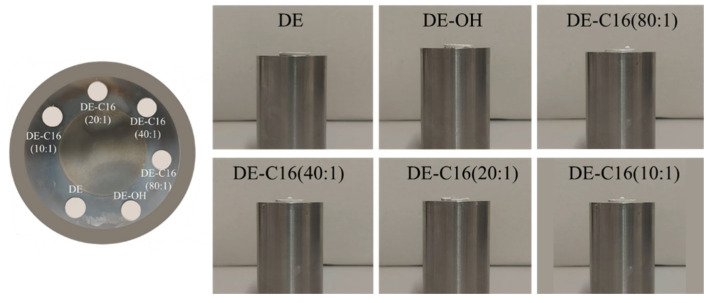
Surface wettability characterization of DE samples. (**Left**) Top view of pristine DE, activated DE-OH, and C16-functionalized DE pellets compacted for testing. (**Right**) Lateral view images of the water droplet behavior on the different substrates. While water was immediately adsorbed by the untreated DE and DE-OH samples, the functionalized samples (from 80:1 to 10:1 molar ratios) demonstrated a significant transition to a hydrophobic state.

The surface wettability of the samples was evaluated to confirm the successful grafting of C16 onto the DE surface. It was observed that for both the pristine and the alkaline-activated DE samples, water droplets were instantaneously adsorbed upon contact, preventing any formal contact angle measurement due to the high hydrophilicity of the material. This behavior highlights the necessity of the silanization process to modify the surface energy. In contrast, all C16-functionalized samples exhibited a distinct hydrophobic character. Figure 4 on the left illustrates the experimental setup, showing the samples compressed into disks and the corresponding lateral views used for image analysis (on the right). The resulting contact angle values are summarized in Table 1.

**Figure 5:**
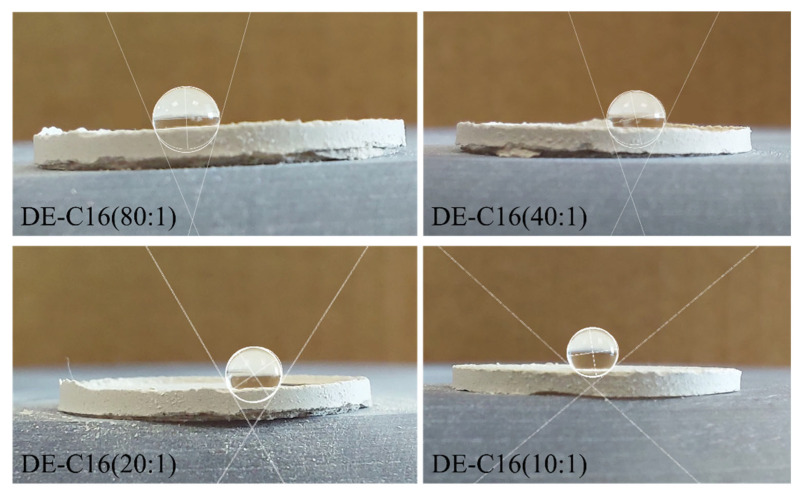
Images of water droplets on C16@DE surfaces at varying concentration ratios (80:1, 40:1, 20:1, and 10:1). The white geometric overlays represent the mathematical fitting of the droplet profiles to determine the contact angle.

To determine the contact angle, the software fits the droplet’s profile using mathematical models, as the spherical and elliptical approximation. This is highly accurate for small droplets (usually <5 μ) where gravity has a negligible effect compared to surface tension. ImageJ software measures the resulting contact angle internally (through the liquid phase) between the solid surface and the tangent line. The data shown in Table 1 indicate a clear correlation between the silane concentration and the degree of hydrophobicity. The contact angle, shown in Figure 5, increases proportionally with the C16 content in the composite. For the sample with the highest silane loading (C16@DE 10:1), the contact angle reached its maximum value, significantly exceeding the thresholds for standard hydrophobicity. This trend suggests that higher molar ratios of C16 lead to a more densely packed organic monolayer of hexadecyl chains. These long alkyl chains effectively shield the polar silanol groups of the DE, transitioning the material’s surface from a super-wetting state to a highly hydrophobic regime.

The maximum contact angle of 134.8° achieved with the 10:1 C16 ratio aligns with previous findings suggesting that long-chain silanes (≥C12) are necessary to promote the molecular ordering and packing density required for high hydrophobicity [16,25]. This transition from a super-wetting state to a robust hydrophobic regime confirms the successful shielding of polar silanol groups [10].

**Figure 6:**
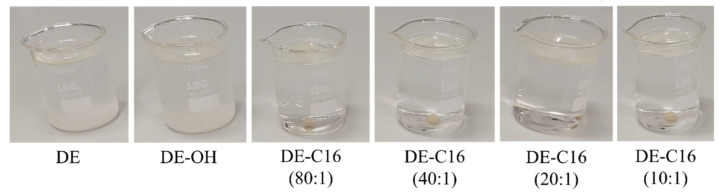
Pictures of visual assessment of the diesel remediation process using different DE variants. Each beaker contains 100 mL of water mixed with 400 μL of diesel fuel and 200 mg of the respective DE powder.

The oil remediation efficiency of C16@DE was initially evaluated through visual qualitative analysis. As illustrated in Figure 6, the introduction of DE powders into a water–diesel mixture reveals distinct interfacial behaviors depending on the surface functionalization. While pristine and alkali-activated DE distribute homogeneously and disperse throughout the aqueous volume due to their hydrophilic nature, the C16@DE variants (at all concentration ratios) exhibit strong hydrophobicity. Initially, these powders float on the water surface before organizing into discrete, compact granules that settle at the bottom of the vessel, effectively encapsulating the majority of the diesel fuel. The pelletization behavior described for diesel adsorption by the C16@DE material, in contrast to the non-functionalized DE and DE-OH, is demonstrated in the Supplementary Materials (Videos S1–S3).

To address the novelty of our findings in relation to literature, it is important to distinguish this work from previous studies on silanized DE. While references [15,16] primarily investigate DE as a functional filler for polymer matrices or as a static superhydrophobic coating on solid substrates, our research focuses on its application as a standalone remediation agent in bulk water. The observed ‘pelletization’ mechanism represents a significant departure from conventional aggregation or flocculation processes. Unlike standard flocculation, which often yields unstable sludges, the C16@DE composites undergo a spontaneous self-organization driven by the minimization of interfacial energy upon contact with hydrocarbons. This process results in the formation of discrete, compact granules that encapsulate the diesel fuel within both the biogenic frustules and the interstitial voids between grains. This mechanism facilitates the direct mechanical recovery of the pollutant-laden material without the need for secondary filtration or complex support systems, providing a distinct practical advantage over previously reported DE-based coatings.

To accurately describe the oil retention mechanism, it is necessary to distinguish between surface-level adsorption and bulk encapsulation. The process in C16@DE is a multi-scale phenomenon, where, initially, hydrocarbon molecules are adsorbed onto the functionalized surfaces via hydrophobic interactions between the grafted C16 alkyl chains and the non-polar diesel components. This is immediately followed by a spontaneous pelletization, where individual silanized grains aggregate into stable granules. These aggregates could create additional interstitial spaces between the grains that, alongside the intrinsic hierarchical porosity of the diatom frustules, provide a massive geometric volume for the physical entrapment of the fuel. This complex ‘synergistic entrapment’ could explain the transition from a dispersed powder to oil-laden pellets.

The driving force behind this pelletization-driven capture is the interplay between the engineered surface wettability and the multiscale porosity of the DE. Upon C16 functionalization, the high-water contact angle (134.8°) generates significant negative capillary pressure for the aqueous phase, effectively preventing water from intruding into the hierarchical micro-nano pores [1,16,22]. Conversely, the low-energy organic layer provides a high affinity for the non-polar diesel molecules, and when they contact the C16@DE grains a strong capillary suction is established [15,25]. This force rapidly draws the hydrocarbon phase into both the intrinsic frustule voids and the newly formed interstitial spaces within the aggregates [11,14]. This selective capillary uptake, enabled by the transition to a hydrophobic/organophilic regime, acts as a “capillary pump” that concentrates the fuel within the granules, stabilizing the pellets and ensuring that the encapsulated oil is not displaced by water during mechanical recovery.

**Figure 7:**
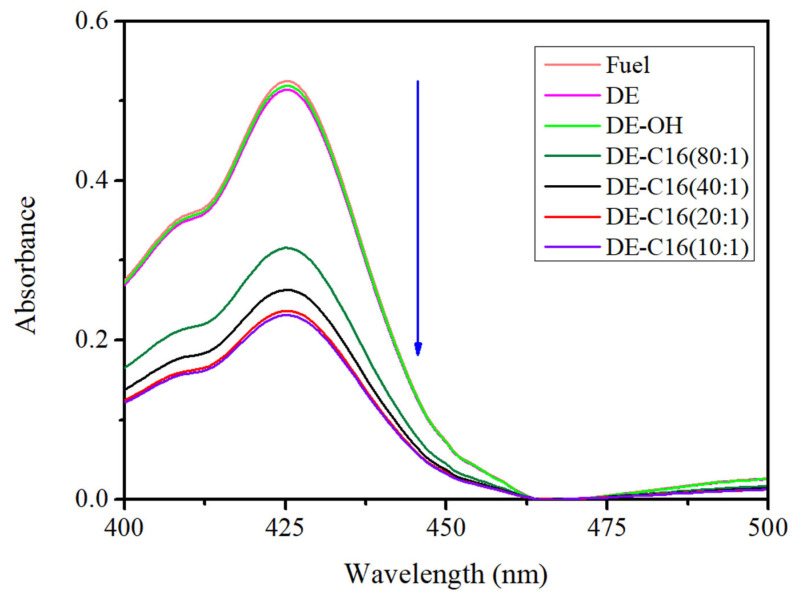
UV/vis absorption spectra of residual diesel fuel following remediation with different DE samples. The orange line represents the reference spectrum of the total initial diesel (400 μL) diluted in 5 mL of benzene. The spectra for pristine DE and alkali-activated DE (DE-OH) are nearly superimposable with the reference, indicating negligible oil uptake. In contrast, the functionalized samples show a significant reduction in absorbance. The blue arrow highlights the progressive decrease in absorbance as the proportion of C16 functionalization increases.

To quantitatively assess the diesel uptake, UV/vis spectrophotometry was employed (Figure 7). Residual diesel, not captured by the DE samples, was extracted into 5 mL of benzene, with the solvent background subtracted for baseline correction. The reference spectrum represents the total initial volume of 400 μL of diesel diluted in 5 mL of benzene. To ensure the accuracy of this method, we first validated the benzene extraction efficiency on control water–diesel samples (without DE), obtaining a practically quantitative recovery (near 100%). Based on this high recovery rate, the amount of oil successfully removed from the water by the functionalized DE was calculated by subtracting the residual oil found in the treated water from the initial oil concentration. This confirms that the reduction in hydrocarbon content is directly attributable to the adsorption and ‘pelletization’ mechanism of our hybrid material. The experimental data confirms that non-functionalized and alkali-activated DE show negligible adsorption, with spectra nearly identical to the reference. In contrast, the C16@DE samples demonstrate significant remediation capabilities. The C16@DE (80:1) sample adsorbed approximately 40% of the fuel, increasing to 50% for C16@DE (40:1) and 55% for C16@DE (20:1). Notably, the C16@DE (10:1) sample showed no significant increase in adsorption compared to the 20:1 ratio, suggesting that the system reached a saturation point regarding the adsorbent’s capacity for the diesel phase.

This sequestration efficiency of approximately 55–56% positions C16@DE as a competitive alternative to other modified diatomite coatings recently explored for oil–water separation, with the added benefit of avoiding complex filtration steps due to the spontaneous pelletization observed [1,28].

To facilitate a direct comparison with existing literature, the gravimetric adsorption capacity of C16@DE (10:1) was calculated. Given the initial diesel load (approx. 0.336 g) and the adsorbent dosage (0.2 g), the 56.3% removal efficiency translates to an adsorption capacity of 0.95 g_oil_/g_adsorbent_. While some specialized synthetic polymers or magnetic alginate/DE beads report higher gravimetric capacities in pure oil phases, our material’s performance is highly significant for a standalone mineral-based system. The primary advantage of C16@DE lies in its ability to combine this capacity with a spontaneous pelletization mechanism, which concentrates the pollutant into stable granules for easy mechanical recovery from contaminated water.

Beyond mineral-based materials, C16@DE offers distinct advantages over conventional polymer sorbents (e.g., polyurethane or melamine sponges) and commercial oil sorbents (e.g., polypropylene mats) [24,35]. While synthetic polymers often exhibit higher theoretical gravimetric capacities, they frequently require energy-intensive synthesis and can face challenges regarding buoyancy loss or secondary pollution through microplastic fragmentation. In contrast, C16@DE utilizes a low-cost biogenic scaffold that maintains structural integrity. Furthermore, unlike commercial sorbents that often act as static filters or require manual retrieval of large, saturated mats, our material’s spontaneous ‘pelletization’ enables a dynamic remediation approach in which the powder can be dispersed over a contaminated area and then mechanically recovered as discrete granules, providing a more versatile and scalable solution for real-world oil spill scenarios.

The quantity of diesel fuel adsorbed by the various DE samples was quantified based on the variation in absorbance relative to the reference spectrum. These results, alongside the specific mass of C16 grafted onto the 200 mg of powder used in each experiment, are summarized in Table 2.

**Figure 8:**
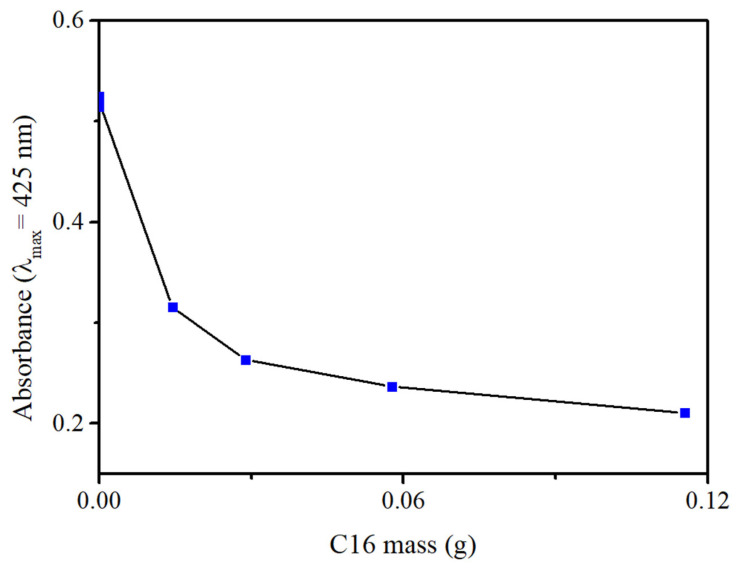
Relationship between C16 mass and absorbance at λ_max_ = 425 nm.

The progressive decrease in absorbance observed in the UV/vis spectra confirms enhanced fuel encapsulation as the functionalization ratio increases. This relationship is further elucidated in Figure 8, where the absorbance intensity at the λ_max_ of 425 nm is plotted against the mass of C16 contained within the composite adsorbents. This plot clearly highlights the system’s transition toward a plateau; as the concentration of C16 increases, the diesel uptake improves significantly until the 20:1 ratio, after which the 10:1 sample demonstrates nearly identical performance. This behavior indicates that the system reaches a saturation point, where additional hydrophobic functionalization no longer provides extra capacity for diesel entrapment under these experimental conditions.

The modification of DE significantly alters its physical properties, as evidenced by the density transitions observed in Table 3. Starting from the base material (DE), which exhibits the lowest density at 0.28 g/mL, the initial hydroxylation or surface treatment leads to a substantial increase in density to 0.77 g/mL in the DE-OH sample. This nearly threefold increase suggests that the treatment effectively modifies the surface or fills the internal pores of the DE, resulting in a more compact and heavier material per unit volume.

Interestingly, subsequent functionalization with C16 chains introduces a downward trend in density that correlates with the loading ratio. As the proportion of C16 molecules relative to the substrate increases, represented by the transition from an 80:1 ratio down to 10:1, the density steadily decreases from 0.67 g/mL to 0.44 g/mL. This inverse relationship is characteristic of hydrophobic bulking in materials science; as long-chain hydrocarbons are grafted onto the surface, they create steric bulk that increases the specific volume of the powder, thereby lowering the packing density compared to the DE-OH precursor.

The observed reduction in bulk density upon increasing silane loading is a clear manifestation of ‘hydrophobic bulking’; as the C16 chains are grafted, the increased steric bulk and specific volume prevent the dense packing observed in the activated DE-OH precursor, a phenomenon critical for maintaining the material’s buoyancy during oil capture [15,16].

Regarding the textural evolution of the material, the literature data for pristine DE report a specific surface area of approximately 3.8 m^2^/g [14]. Upon functionalization, the grafting of long alkyl chains is established to cause a progressive reduction in both the BET specific surface area and the total pore volume as the silane moieties partially occupy or clog the hierarchical micro-nano pores. In our study, this textural modification is indirectly but consistently validated by the density analysis (Table 3). The steady decrease in bulk density from 0.77 g/mL (DE-OH) to 0.44 g/mL (C16@DE 10:1) provides a quantitative measure of the “hydrophobic bulking” and space-filling effect exerted by the dense C16 layer within the biogenic scaffold, which effectively alters the material’s porosity and surface energy.

## 4. Conclusions

This research successfully demonstrates a robust and scalable methodology for transforming naturally hydrophilic DE into a high-performance hydrophobic adsorbent tailored for environmental remediation. This transformation was achieved through a systematic two-step functionalization process, wherein alkaline activation was employed to maximize reactive surface sites, followed by the covalent grafting of C16. The resulting chemical modification effectively altered the surface energy of the material; while pristine and activated DE exhibited hydrophilic properties, the C16-treated samples demonstrated a dramatic shift toward robust hydrophobicity, reaching a maximum static contact angle of 134.8° at the 10:1 DE:C16 molar ratio. The functionalization process significantly influenced the physical properties of the composites, notably through a “hydrophobic bulking” effect. While alkaline activation initially increased the material density to 0.77 g/mL by modifying the surface, subsequent silanization introduced long-chain hydrocarbons that created steric bulk, ultimately lowering the bulk density to as little as 0.44 g/mL. Beyond these structural changes, the functionalized materials showed a significant capacity for diesel fuel encapsulation. Unlike pristine DE, which disperses homogeneously in water, the C16@DE composites exhibit a spontaneous self-organization into discrete pellets upon contact with hydrocarbons. This unique “pelletization” mechanism effectively traps the pollutant within stable granules, facilitating its mechanical recovery from aquatic environments. Quantitative UV/vis spectrophotometric analysis confirmed the efficiency of this remediation pathway, revealing that the C16@DE systems can sequester approximately 55–56% of the diesel phase from contaminated water. A clear correlation was established between the mass of the C16 grafting agent and the resulting adsorption capacity, identifying a critical system saturation point at the 20:1 DE:C16 molar ratio. Because increasing the silane loading to a 10:1 ratio yielded no significant improvement in oil uptake, the 20:1 ratio represents the optimal efficiency plateau for this adsorbent, at least in the C16:DE ratios here explored. This study offers a promising innovation by repurposing low-cost, biogenic silica into a specialized tool for environmental protection. These findings position functionalized DE as a sustainable, eco-friendly, and cost-effective potential solution for addressing marine oil spills and industrial wastewater treatment. Future research will focus on the regeneration and reusability of these functionalized composites to further enhance their economic and environmental value. While the unique “pelletization” mechanism already allows for the mechanical recovery of diesel-laden granules from aquatic environments, subsequent studies should investigate efficient pathways for hydrocarbon extraction, such as solvent washing or controlled thermal desorption, to restore the adsorbent’s capacity. Evaluating the performance of C16@DE over multiple sequestration cycles will be a crucial step toward validating its industrial applicability and ensuring a cost-effective, circular approach to marine oil spill remediation and wastewater treatment.

## Figures and Tables

**Table 1:** Static contact angle measurements and associated uncertainties for C16-functionalized DE samples.

Sample	Contact Angle (°)	Uncertainty	Circle St.Dev	Ellipse St.Dev
DE_C16 (80:1)	98.3	0.9	1.668	6.888
DE_C16 (40:1)	103.5	2.4	1.107	4.201
DE_C16 (20:1)	117.6	2	1.219	3.427
DE_C16 (10:1)	134.8	0.6	1.220	6.976

**Table 2:** Quantitative analysis of fuel remediation efficiency.

Sample	C16 Mass (g)	Fuel Adsorption (%)
C16@DE (80:1)	0.0144 ± 0.0021 ^1^	40.4 ± 1.2 ^1^
C16@DE (40:1)	0.0288 ± 0.0035	50.3 ± 3.2
C16@DE (20:1)	0.0577 ± 0.0028	55.2 ± 1.7
C16@DE (10:1)	0.1154 ± 0.0044	56.3 ± 2.2

^1^ ±Std.dev. Calculating the average over 3 independent measurements.

**Table 3:** Density of samples (25 °C).

Sample	ρ (g/mL)
DE	0.28 ± 0.02 ^1^
DE-OH	0.77 ± 0.07
C16@DE (80:1)	0.67 ± 0.08
C16@DE (40:1)	0.56 ± 0.04
C16@DE (20:1)	0.51 ± 0.05
C16@DE (10:1)	0.44 ± 0.07

^1^ ±Std.dev. Calculating the average over 10 independent measurements.

## Data Availability

The data presented in this study are openly available in Zenodo at DOI:10.5281/zenodo.18937885.

## Supplementary Materials

The following supporting information can be downloaded at: https://zenodo.org/uploads/18668149 (accessed on 17 February 2026), Video S1: water–diesel + DE; Video S2: water–diesel + DE-OH; Video S3: water–diesel + C16@DE(80_1).

## Abbreviations

The following abbreviations are used in this manuscript:

ATR-FTIR	Attenuated Total Reflection Fourier Transform Infrared
C16	Hexadecyltrimethoxysilane
DE	Diatomaceous Earth
EtOH	Absolute ethanol
SEM	Scanning Electron Microscope
UV/vis	Ultraviolet visible
